# Effects of dietary curcumin or N-acetylcysteine on NF-κB activity and contractile performance in ambulatory and unloaded murine soleus

**DOI:** 10.1186/1743-7075-2-20

**Published:** 2005-08-26

**Authors:** Mehran Farid, Michael B Reid, Yi-Ping Li, Eric Gerken, William J Durham

**Affiliations:** 1 Pulmonary Medicine, Baylor College of Medicine, One Baylor Plaza, Houston, TX, 77030, USA; 2 Department of Physiology, College of Medicine, University of Kentucky, Lexington, KY,40506, USA

## Abstract

**Background:**

Unloading of skeletal muscle causes atrophy and loss of contractile function. In part, this response is believed to be mediated by the transcription factor nuclear factor-kappa B (NF-κB). Both curcumin, a component of the spice turmeric, and N-acetylcysteine (NAC), an antioxidant, inhibit activation of NF-κB by inflammatory stimuli, albeit by different mechanisms. In the present study, we tested the hypothesis that dietary curcumin or NAC supplementation would inhibit unloading-induced NF-κB activity in skeletal muscle and thereby protect muscles against loss of mass and function caused by prolonged unloading.

**Methods:**

We used hindlimb suspension to unload the hindlimb muscles of adult mice. Animals had free access to drinking water or drinking water supplemented with 1% NAC and to standard laboratory diet or diet supplemented with 1% curcumin. For 11 days, half the animals in each dietary group were suspended by the tail (unloaded) and half were allowed to ambulate freely.

**Results:**

Unloading caused a 51–53% loss of soleus muscle weight and cross-sectional area relative to freely-ambulating controls. Unloading also decreased total force and force per cross-sectional area developed by soleus. Curcumin supplementation decreased NF-κB activity measured in peripheral tissues of ambulatory mice by gel shift analysis. In unloaded animals, curcumin supplementation did not inhibit NF-κB activity or blunt the loss of muscle mass in soleus. In contrast, NAC prevented the increase in NF-κB activity induced by unloading but did not prevent losses of muscle mass or function.

**Conclusion:**

In conclusion, neither dietary curcumin nor dietary NAC prevents unloading-induced skeletal muscle dysfunction and atrophy, although dietary NAC does prevent unloading induced NF-κB activation.

## Background

Prolonged exposure to microgravity causes muscle atrophy, resulting in weakness and predisposition to fatigue. Such changes can markedly degrade astronauts' performance, especially upon return to Earth [1]. Atrophy starts as early as 4 days into space flight [2] and becomes more significant in long-term space missions [3]. In addition to atrophy, weight-bearing skeletal muscles lose their contractile force per cross-sectional area [4]. Since atrophy in space flight and disuse atrophy share similar pathophysiologic mechanisms [5], countermeasures to microgravity-induced muscle wasting may also be effective for the prevention of disuse atrophy and muscle weakness in long term bedridden patients. Thus, there is considerable interest in the development of countermeasures that oppose the complications of muscle disuse.

Hindlimb unloading, a commonly used model to study the effects of muscle disuse, activates the transcription factor nuclear factor-κB (NF-κB) in weight-bearing muscles [6,7]. This laboratory has recently reported that NF-κB activation increases ubiquitin conjugation [8], consistent with a role for NF-κB in the regulation of skeletal muscle protein breakdown [9]. In addition, activation of NF-κB in response to TNF-α alters myogenesis [10] and is essential for the loss of protein in TNF-α-stimulated skeletal muscle myotubes [11]. Stimuli that persistently activate NF-κB are associated with muscle protein loss and activation of the ubiquitin-proteasome pathway [12]. In vitro, selective inhibition of NF-κB can prevent loss of muscle protein [11]. In vivo, a recent study found that mice lacking specific NF-κB family members (p105/50 or bcl3) are resistant to unloading-induced atrophy and NF-κB activation [6], supporting a role for NF-κB as a robust in vivo catabolic stimulus during disuse atrophy.

The prevention of unloading-induced atrophy by ablation of genes for specific NF-κB family members [6] suggests that pharmacological inhibition of NF-κB might also reduce atrophy in response to unloading. In the present study, we tested the ability of two NF-κB inhibitors to inhibit disuse-induced skeletal muscle NF-κB activation. Curcumin (diferuloylmethane), a major component of the spice turmeric (Curcuma longa), has been reported to exert in vivo effects on serum lipids [13], aortic atherosclerosis [14], and various forms of cancer [15]. Curcumin also possesses anti-inflammatory [16] and antioxidant [14] properties in vivo and inhibits the canonical NF-κB activation pathway [17]. In the canonical activation pathway, the NF-κB inhibitor IκB is phosphorylated by IκB kinase (IKK), leading to its dissociation from NF-κB and subsequent translocation of NF-κB to the nucleus [6], where it binds to the promoter region of target genes. Curcumin acts at a proximal step in the canonical activation pathway, inhibiting IKK [17] phosphorylation of IκBα. N-acetylcysteine (NAC) is a naturally occurring compound found in several vegetables, including garlic [18], onion [18], peppers [19], and asparagus [19]. In addition to antioxidant, antiangiogenic, and anticarcinogenic properties [20], NAC inhibits NF-κB activation by several mechanisms acting distal to the activity of IKK [21-23].

NF-κB activation in response to muscle disuse has been reported to occur through a pathway distinct from the canonical activation pathway that does not involve IκB activation [6]. In the present study, we tested the hypothesis that curcumin, an inhibitor of the canonical activation pathway, and NAC, acting downstream of curcumin in the NF-κB activation pathway, would inhibit NF-κB and preserve muscle mass and function. In addition, as the in vivo effects of dietary curcumin on NF-κB activity are not well-studied, particularly under basal (non-induced) conditions, we measured NF-κB activity in liver, soleus, lung, and large intestine to test the hypothesis that dietary curcumin inhibits basal NF-κB activity in vivo.

## Methods

### Animals

Adult male ICR mice were purchased from Harlan (Indianapolis, IN) and housed under pathogen-free conditions. Animals were handled in accordance with the policies and guidelines for the care and use of laboratory animals of the National Institutes of Health. The protocol was approved by the Animal Protocol Review Committee at Baylor College of Medicine.

### Food

Mice in control and treatment groups were fed a standard chow diet (Purina-Mills, 5053 diet, Labdiet, St Louis, MO). For the curcumin treatment group, the diet was modified by adding commercial curcumin (LKT laboratories, St. Paul, Minnesota) to 1% (w/w). Curcumin was uniformly incorporated into the food, which was pelleted to ensure uniform feed and curcumin intake. All control and experimental diets were stored at 4°C. All mice had free access to water during the dietary treatment period. For the NAC treatment group, 1% NAC (w/v) was included in the drinking water.

### Methods

**Figure 1:**
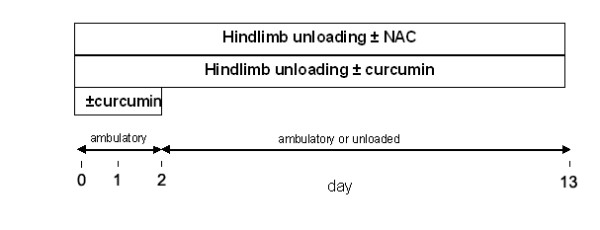
**Unloading protocol**. In study 1, ambulatory mice were fed either a control or a 1% curcumin diet for 2 days. In study 2, mice were divided into control and curcumin diet groups as in study 1 for the first two days, after which each diet group was further divided into ambulatory or unloaded groups.

In the first study (Figure 1), ambulatory mice were studied after two days on either the control diet or the curcumin-supplemented diet, in order to test whether curcumin inhibited NF-κB in vivo. In the second study (Figure 1), the effects of curcumin were studied in three groups of mice. Two groups received the control diet throughout the 13-day study period, whereas the other group received the curcumin diet. After 2 days, the curcumin dietary group and one of the groups receiving the control diet were hindlimb-unloaded. In the two unloaded groups, the tail of each animal was taped to suspension wire using first aid tape in order to elevate the hindlimb of each animal. Mice remained unloaded for the remainder of the study period. Unloaded mice were able to ambulate using their forelegs; however, their hindlimbs were unable to touch the bottom or sides of the cage. In the third study (Figure 1), three groups were studied: one ambulatory group receiving normal drinking water, one hindlimb-unloaded group receiving normal drinking water, and one unloaded group receiving 1% NAC in the drinking water. All mice in the third study received the standard chow diet.

### Tissue collection

Mice were deeply anesthetized by methoxyflurane inhalation and soleus muscles were excised with their tendons intact. Following collection of the solei, animals were killed by cervical dislocation. In the first study, samples from the lungs, liver, and distal colon were taken immediately post-sacrifice. In the second and third studies, only solei were collected. All tissues were rinsed in Krebs-Ringer solution containing (mM) 137 NaCl, 5 KCl, 1 NaH_2_PO_4_, 24 NaHCO_3_, 2 CaCl_2_, and 1 MgSO_4 _and gently blotted dry prior to weighing and freezing. The solution was maintained at room temperature and aerated with 95% O_2 _+ 5% CO_2_. In the second and third studies, solei were used for contractile studies prior to weighing and freezing (see below).

### Contractile protocol

For contractile studies, one tendon of the soleus muscle was fixed to a glass bar with silk suture (size 5-0); the other tendon was tied to a force transducer (model BG 100, Kulite Instruments, Leonia, NJ) mounted on a micrometer. Solei were stimulated by field stimulation with platinum electrodes. Muscle length was adjusted to generate maximal twitch force (optimal length, L_0_). The muscle bath was warmed to 37°C after determination of L_o_. This temperature was maintained by digital water bath throughout the remainder of the experiment. After a 30-min thermoequilibration period, twitch characteristics (including twitch force, time to peak force, and half-relaxation time) were measured, followed by determination of the force frequency relationship. To determine the force frequency relationship, tetanic contractions were stimulated at 2 min intervals (500-ms train duration, 17 volts). Between each intermediate frequency (15, 30, 50, 80, 120, 160, 250 Hz), a maximum tetanic contraction (P_0_, 300 Hz) was elicited to serve as a reference for changes in force over time. One minute after the last stimulation, the muscle was subjected to a fatiguing contraction protocol by stimulating the muscle at 40 Hz using a 1:4 duty cycle (0.5 trains/s, 500-ms train duration). Passive and developed forces were recorded on a strip chart for later analysis. At the end of the experiment, soleus length and weight were measured. Force measurements were normalized for functional cross section according to Close [24]. Results from contractile studies of mice receiving the control diet have been previously reported [7].

### Preparation of tissue extracts

Cytoplasmic extracts were prepared by grinding tissue samples in a solution containing 10 mM HEPES-KOH (pH 7.9), 1.5 mM MgCl_2_, 10 mM KCl, 2 μg/ml aprotinin, 1 μg/ml leupeptin, 1 mM DTT, and 2 mM PMSF. Samples were ground in a glass tissue grinder cooled in an ice water slurry. After grinding, samples were vortexed, incubated on ice for ≥ 10 minutes, vortexed again, and then frozen on dry ice. After thawing in cool water, samples were vortexed a final time and centrifuged for 10 s at 4°C. The supernatant, containing the cytosolic proteins, was then collected for subsequent analyses. Nuclear extracts were prepared by resuspending the pellet in a buffer containing 20 mM HEPES-KOH (pH 7.9), 1.5 mM MgCl_2_, 5% glycerol, 420 mM NaCl, 0.2 mM EDTA, 2 μg/ml aprotinin, 1 μg/ml leupeptin, 1 mM DTT, and 2 mM PMSF, and incubating for 30 minutes at 4°C. During incubation, samples were vortexed ~10 s every 10 min. Following incubation, the samples were centrifuged 4 minutes at 4°C and the supernatant, containing the nuclear proteins, was collected.

### Protein determination

For determination of cytosolic and nuclear protein concentrations, 999 μl of a 1:4 mixture of Bio-Rad concentrated dye reagent (#500-0006, Bio-Rad, Hercules, CA) and water were added to 1 μl of extract. The sample was vortexed and the absorbance at 595 nm was measured. Protein concentration was determined using a standard curve of absorbance vs. concentration of bovine serum albumin (BSA). Samples and standards were analyzed in triplicate.

### Electrophoretic mobility shift assay (EMSA)

Binding reactions were performed using 3–10 μg nuclear protein combined with 1 ng of NF-κB-binding DNA probe (5'AGTTGAGGGGACTTTCCCAGGC-3'), labeled with [α-^32^P]dATP (Amersham-Pharmacia) using the Klenow fragment. Binding reactions were carried out in binding buffer containing 10 mM Tris·HCl (pH 7.5), 10% glycerol, 0.05% NP-40, 0.1 μg/μl BSA, 0.05 μg/μl poly (dI-dC), and 500 μM DTT. Samples were incubated on ice for 30 minutes. Supershifts with antibodies to the p50 and p65 subunits of NF-κB (Santa Cruz; Santa Cruz, CA), were added 15 minutes into the incubation. After incubation, samples were resolved on 5% polyacrylamide gels. After drying, gels were exposed to x-ray film and analyzed using commercial densitometry software (ImageQuant 5.2, Amersham Biosciences Corp., Piscataway, NJ). Results from studies of mice receiving the control diet have been previously reported [7].

### Statistical analysis

Values are means ± SE. A one-way ANOVA (diet × frequency or diet × time) was used to compare force production among groups at different stimulation frequencies or time-points. For differences in single measurements in paired groups we utilized paired t-tests. P value was set < 0.05. Statistical analyses were carried out using SigmaStat, version 3.00.

## Results

### Body weight and diet

The curcumin and NAC treatments were well-tolerated by the mice; no overt changes in behavior, grooming, or response to handling were observed in the treated mice. To verify that the curcumin diet had systemic effects on NF-κB activity, as well as to screen for gross adverse effects of the curcumin diet, in the first study we measured protein concentrations and NF-κB activity in a panel of tissues after 2 days of feeding the curcumin-supplemented diet. Curcumin feeding did not affect cytosolic or nuclear protein concentrations in any of the tissues tested (data not shown). In addition, after 48 hours of feeding, neither absolute (control: 1.65 ± 0.11 g, curcumin: 1.67 ± 0.07 g) nor relative (control: 54 ± 3 mg/g body weight, curcumin: 56 ± 2 mg/g body weight) liver weight was affected by curcumin (P > 0.05).

In a second study, ambulatory and unloaded mice were fed either the control diet or the curcumin diet. Ambulatory mice gained weight, regardless of whether they received the curcumin or the control diet, whereas the weight of the unloaded mice in both diet groups did not change over the course of the experiment (Table 1). There was no effect of diet on animal weight. Unloading reduced water intake (Table 1), whereas diet had no effect on this variable (Table 1).

**Table 1:** Dietary intakes and body weights.

	**Curcumin study**			**NAC study**		
**Experimental groups (diet)**	**Ambulatory Control**	**Unloaded Control**	**Unloaded Curcumin**	**Ambulatory Control**	**Unloaded Control**	**Unloaded Curcumin**

Number of mice	11	11	11	3	6	7
Body weight, Day 0	26.5 ± 0.8	26.8 ± 0.7	27.3 ± 0.9	22.8 ± 1.5	24.1 ± 1.0	23.1 ± 0.8
Day 11	30.0 ± 1.2 *	26.3 ± 0.7	26.5 ± 0.8	28.4 ± 1.5	21.4 ± 0.8	20.7 ± 0.7
NAC intake (mg/g/day)	-	-	-	-	-	0.78 ± 0.04
Total curcumin intake (g/kg)	-	-	22 ± 2		-	-

In the third part of this study, unloaded mice receiving either water or NAC (1% w/v) in the drinking water were compared with ambulatory mice receiving water. As in the curcumin study, ambulatory mice gained weight during the study period whereas unloaded mice neither gained nor lost weight, regardless of treatment (Table 1). Unloaded mice receiving NAC in their drinking water consumed less water than unloaded mice receiving water alone (data not shown).

#### Effect of curcumin on basal NF-κB activity

**Figure 2:**
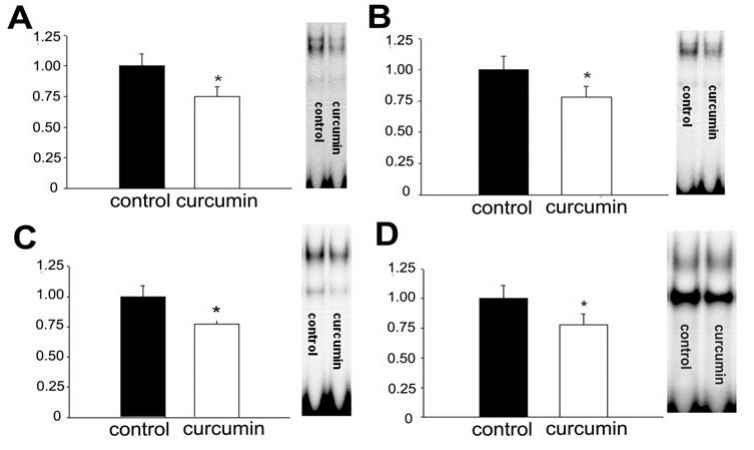
**Effect of 1% curcumin diet on basal NF-κB activity in various tissues**. Mice were fed a 1% curcumin diet for 2 days and then sacrificed for measurement of NF-κB activity. (*P < 0.05 vs control, N = 6–8 comparisons). The right panel for each graph depicts results from a typical gel shift assay. A) Liver, B) Soleus, C) Lung, D) Large Intestine.

To evaluate the in vivo effects of oral curcumin ingestion on NF-κB activity in different tissues, in the first study we measured NF-κB activity in intestine, liver, lung, and soleus of ambulatory mice fed either the curcumin diet or the control diet for 2 days. Basal NF-κB activity was readily apparent in all of the tissues analyzed (Fig. 2). The curcumin diet reduced basal NF-κB activity by 22–25% in all tissues tested (Fig. 2).

#### Effects of unloading and diet on soleus muscle

**Figure 3:**
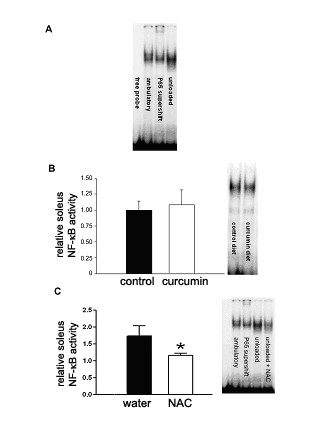
**Effect dietary treatment on NF-κB activity in unloaded soleus**. Panel A. Typical gel shift results showing effect of unloading on NF-κB activity. Panel B. Curcumin effects on NF-κB activity in unloaded soleus muscle. The right panel depicts results from a typical gel shift assay. Panel C. NAC effects on NF-κB activity in unloaded soleus muscle. The right panel depicts results from a typical gel shift assay.

Unloading reduced soleus weight by ~40–50%, with no effect of either curcumin or NAC on this response (Table 2). Cross sectional area of the soleus was also smaller in unloaded mice than in ambulatory mice; neither curcumin nor NAC modulated this response (Table 2). Soleus NF-κB activity was not different in unloaded mice receiving curcumin and those receiving the normal diet (Fig. 3). In contrast, oral NAC completely prevented the unloading-induced increase in soleus NF-κB activity (Fig. 3). Neither unloading nor diet altered protein concentrations in the soleus (data not shown).

**Table 2:** Effects of conditioning and treatment on muscle properties.

	**Ambulatory**		**Unloaded**			
**Parameter**	**Control**	**Curcumin**	**Control**	**Curcumin**	**Control**	**NAC**

*Soleus weight (mg)*	7.3 ± 0.3	6.9 ± 0.4	3.4 ± 0.0^a^	3.3 ± 0.2^a^	3.6 ± 0.1^a^	3.8 ± 0.2^a^
*Muscle length (cm)*	1.10 ± 0.02	1.13 ± 0.02	1.02 ± 0.03^b^	1.09 ± 0.03^b^	0.96 ± 0.03	0.92 ± 0.03
*Cross sectional area (cm^2^)*	6.3 ± 0.0	5.8 ± 0.0	3.2 ± 0.0^c^	3.0 ± 0.0^c^	3.5 ± 0.1	3.9 ± 0.1

### Effects of unloading and dietary treatment on contractile performance

**Figure 4:**
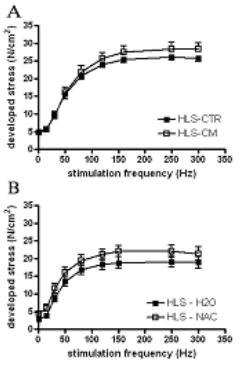
**Force frequency relationship in mouse solei**. *A*. Curcumin effects on force-frequency relationship in unloaded mice. *B*. NAC effects on force-frequency relationship in ambulatory and unloaded mice treated with either the control or the NAC-supplemented diet. Values are mean ± SE of force per unit area (N/cm^2^).

Hindlimb unloading reduced force production; neither curcumin nor NAC prevented this response (Fig. 4). Twitch stress, time to peak, and half relaxation time, as well as fatigue, were similar in all groups (data not shown).

## Discussion

NF-κB activation is associated with stimulation of the ubiquitin-proteasome pathway and loss of skeletal muscle protein [6,8,12], suggesting that unloading-induced NF-κB activation may contribute to the catabolic response to muscle unloading [6]. In the present study, we tested the hypothesis that inhibition of NF-κB would prevent unloading-induced skeletal muscle atrophy and weakness. We report two new findings: 1) dietary curcumin, an inhibitor of the classical NF-κB pathway, does not inhibit unloading-induced NF-κB activation, atrophy, or weakness, in agreement with a previous study suggesting unloading-induced NF-κB activation does not occur by the classical pathway [6], and 2) dietary NAC, which inhibits NF-κB activation by both classical and nonclassical pathways, prevents unloading-induced NF-κB activation but does not inhibit unloading-induced atrophy or weakness, suggesting that NF-κB inhibition alone is insufficient to prevent these responses.

A recent study found that unloading-induced atrophy either did not occur or was greatly reduced in mice lacking genes for the NF-κB family members Nfkb1 or Bcl3 [6]. Taken together with the results from the present study, it appears that if it is possible to inhibit unloading-induced atrophy by pharmacological NF-κB inhibition it will require greater or more targeted (e.g. specific for p105/50 and/or bcl3) inhibition than was achieved in the present study using NAC.

Unloading increases skeletal muscle reactive oxygen levels [25,26], which are a stimulus for NF-κB activation [12]. Our finding that NAC prevented unloading-induced NF-κB activation suggests this response is secondary to increased levels of reactive oxygen species. The finding that NAC did not prevent unloading-induced muscle atrophy and contractile dysfunction is consistent with a previous study that found antioxidant supplementation (including NAC) during unloading did not prevent these responses [25].

In addition to stimulating muscle protein breakdown, skeletal muscle unloading inhibits muscle protein synthesis [27,28]. In the present study, inhibition of NF-κB activity by NAC did not prevent the atrophic response to unloading. This may reflect that reduced protein synthesis is the dominant factor responsible for unloading-induced atrophy. It is also possible that NF-κB inhibition alone is not sufficient to prevent unloading induced muscle protein breakdown.

Curcumin is an established inhibitor of NF-κB [17]. However, most of the studies examining the effects of curcumin on NF-κB activity have been performed in vitro. Thus, in the present study we determined the effect of short-term (2 days) dietary curcumin on basal NF-κB activity in soleus, lung, intestine, and liver, to determine whether dietary curcumin had in vivo effects. Curcumin ingestion slightly inhibited basal NF-κB activity in all tissues under basal conditions but did not inhibit the NF-κB activation that occurs in skeletal muscle in response to unloading.

Despite the fact that curcumin did not inhibit unloading-induced NF-κB activation, the finding that basal muscle NF-κB activity was inhibited by curcumin is noteworthy, as NF-κB is emerging as an important regulator of skeletal muscle metabolism. Activation of NF-κB in skeletal muscle myotubes increases ubiquitin conjugation [8], consistent with a role for NF-κB in muscle protein catabolism [9]. NF-κB activation is also thought to contribute to cancer-induced cachexia, in which muscle loss is often dramatic and is a strong predictor of a poor outcome [29]. In addition, recent studies have suggested a possible role for NF-κB in the development of insulin resistance [30].

## Conclusion

In conclusion, this study shows that oral curcumin as a dietary supplement inhibits NF-κB activity similarly in a variety of tissues in ambulatory mice. However, it does not inhibit NF-κB activity in the solei of unloaded mice and, contrary to our original hypothesis, does not prevent atrophy of unloaded soleus muscles. Similarly, oral NAC treatment had no effect on unloading-induced atrophy although, in contrast to curcumin, it did prevent unloading-induced NF-κB activation.

## Competing interests

The author(s) declare that they have no competing interests.

## Authors' contributions

MF carried out the curcumin feeding and contractile studies, contributed to the design of the study, and assisted in the writing of the manuscript. EG carried out the NAC feeding and contractile studies, contributed to the design of the study, and assisted in the writing of the manuscript. MBR conceived of the study and design, contributed to the data interpretation, and participated in manuscript preparation. YPL contributed to data analysis and interpretation and participated in manuscript preparation. WJD performed the protein and gel shift assays, contributed to study design and data interpretation, and participated in manuscript preparation.
